# Could a tuberculosis epidemic occur in London as it did in New York?

**DOI:** 10.3201/eid0601.000102

**Published:** 2000

**Authors:** A C Hayward, R J Coker

**Affiliations:** University of Nottingham, United Kingdom. Andrew.Hayward@Nottingham.ac.uk

## Abstract

In early 1999, more than 160 senior physicians, public health officials, and nurses met to discuss London's tuberculosis (TB) control program. The program was examined against the public health response of New York City's Bureau of Tuberculosis Control during a 1988 to 1992 epidemic. This article outlines TB epidemiology and control in New York City 10 years ago and in London today to assess whether the kind of epidemic that occurred in New York could occur in London.


### Could a Tuberculosis Epidemic Occur in London as It Did in New York?

**Andrew C. Hayward*† and Richard J. Coker**
*University of Nottingham, Nottingham, United Kingdom; and
†St. Mary's Hospital, London, United Kingdom

In early 1999, more than 160 senior physicians, public health officials, and nurses met to discuss London's tuberculosis (TB) control program. The program was examined against the public health response of New York City's Bureau of Tuberculosis Control during a 1988 to 1992 epidemic. This article outlines TB epidemiology and control in New York City 10 years ago and in London today to assess whether the kind of epidemic that occurred in New York could occur in London.

Worldwide, tuberculosis (TB) kills more people than any other infection (1). Prevalence of the disease has been increasing in many countries since the mid-1980s, and more people are ill with TB now than at any other time in history (1). Although most cases occur in the developing world, some major urban areas in industrialized countries have also had a resurgence of the disease (1).

In the late 1980s and early 1990s, New York City had a major epidemic of TB, with rates tripling in 15 years and outbreaks of multidrug-resistant TB in many hospitals. Massive reinvestment in TB services to control the epidemic (2,3) resulted in a 59% decrease in cases, from a peak of >3,700 in 1992 to <1,000 in 1998. The incidence of multidrug-resistant TB decreased by 91% over the same period (4).

In the United Kingdom, TB rates began to increase in 1988 but have since leveled off (5), except in London, where the increase has been much more dramatic and has not subsided (6). This article compares the current epidemiology and control of TB in London with the situation in New York City in the late 1980s. Urgent action is needed to strengthen TB control in London if an epidemic like that in New York City is to be avoided.

Address for correspondence: Andrew C. Hayward, Division of Public Health and Epidemiology, University Hospital, Queen's Medical Centre, Nottingham, NG7 2UH, United Kingdom; fax: 441-115-970-9312; e-mail: Andrew.Hayward@Nottingham.ac.uk.

### Epidemiology of TB in the Two Cities

The increase in TB notifications in London is similar to that seen during the first 10 years of the epidemic in New York City (Figure) (7). As in New York, rates of disease differ considerably in different parts of the city, with the highest rates in areas of low socioeconomic conditions and large immigrant populations (2,3,6). In New York, the highest rates were in central Harlem (79 per 100,000 in 1980 to 170 in 1989) (2). In London, the highest rates are in Newham, Tower Hamlets, and Brent (77-79 per 100,000 population) (6). Rates in several London boroughs have increased two- to threefold in 10 years (6). In both cities, the increase in case reports has

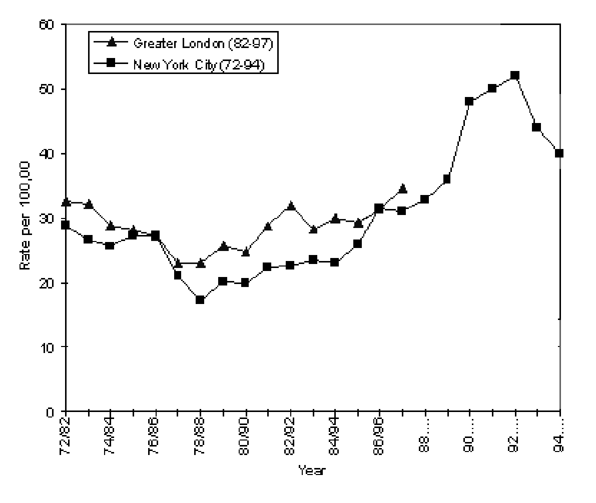

Figure. Tuberculosis rates in London, 1982–1997, compared with those in New York, 1972–1994.

been mainly among young adults (15-24 years of age) (3,6).

In both cities, most cases are in nonwhite residents. In New York City in 1994, 50% of TB patients were classified as black, non-Hispanic; 26% as Hispanic; 12% as white, non-Hispanic; and 12% as other/unknown (8). In London in 1993, 40% were classified under Indian Subcontinent, 31% white, and 29% "other nonwhite" (9). From 1988 to 1993, London rates increased most in the "other nonwhite" category (mainly black Africans) but also increased among whites and nonwhites who were born in the United Kingdom. (The rate among those of Indian Subcontinent background who were born in the United Kingdom rose from 12 per 100,000 in 1988 to 41 per 100,000 in 1993 [9]). Therefore, importation of disease is not the only factor causing the increase in London.

In London (as in New York at the beginning of the epidemic), data on patient origins are not routinely collected. Preliminary data suggest that at least 55% of cases are among foreign-born persons (H. Maguire, pers. comm.). In New York in 1993-94, approximately 28% of TB cases were among immigrants (3). Thus, in both cities a substantial proportion of cases is among immigrants, but this disparity is greater in London.

Early in the epidemic in New York, 38% of persons with culture-confirmed cases of TB were known to be HIV infected (2). Data on HIV infection in TB patients are not routinely collected in the United Kingdom, but a study in 1993 estimated that at least 7% of TB cases in London were among HIV-positive persons (10). A more recent study of 157 patients starting treatment for TB at an inner-city London hospital in 1996 and 1997 showed that 25% were coinfected with HIV (44% Europeans, 49% Africans, and 3% Asians). These data suggest that, in some ethnic groups in some areas of London, HIV infection is strongly affecting the epidemiology of TB (11).

Data on drug resistance were not routinely collected early in the epidemic in New York City. In the early 1990s, 19% of patients with culture-confirmed TB had multidrug-resistant TB, and in 26% of patients TB was resistant to isoniazid (12). These figures had declined to 4.1% and 4.4% by 1998 (4). In 1994-96, 2.6% of isolates in London were multidrug resistant, compared with 1% in the rest of the country; 8% were resistant to isoniazid, compared with 4.7% in the rest of the country (13). The current levels of drug resistance in London are lower than those during the epidemic in New York. However, data from New York demonstrate that transmission of resistant strains in hospitals and other settings can lead to a rapid increase in rates of resistance (14). Several outbreaks of TB, including two involving multidrug-resistant TB, have already occurred in London, principally affecting HIV-positive patients in teaching hospitals (15-17).

Recent transmission appears to be a substantial problem in both cities. Preliminary results from a molecular study to identify recent transmission of TB in inner London suggest that approximately 27% of cases are clustered (10). In New York City, an analysis of the molecular epidemiologic links of TB isolates obtained in April 1991 showed that, of 344 patients, 126 (37%) belonged to one of 31 clusters, and clustering was more frequent in patients who had multidrug-resistant TB (involved in 53% of clusters), were black (44%), and were homeless (49%) (14).

Both London and New York City have large populations of homeless people (86,000 in New York in 1990 compared with 50,000 in London in 1992) (18,19). In New York City in the 1970s and 1980s, approximately 6% to 7% of homeless persons had active TB (20,21). By 1992, up to 25% of the city's cases of TB occurred in the homeless (22). Fewer data are available for TB rates in London's homeless, but information suggests very high levels of TB. Screening at a shelter in London in 1993 identified active TB in 1.5% of those screened. Another 3.5% had chest X-rays suggestive of active disease, but the patients were lost to follow-up before further investigation could take place (23). At least 5% of TB patients in London have a history of residence in a hostel for the homeless (H. Maguire, pers. comm.). In both cities, poor compliance with treatment and transmission of disease are major problems in the homeless (18,21).

TB transmission in state correctional facilities was a major problem in New York (3). No outbreaks of TB have yet been documented in prisons in London.

### Control in London and New York City

The loss of government funding of TB programs in the 1970s and 1980s in New York City made access to treatment more difficult for poorer sectors of the population (often nonwhite patients) (3), likely contributing to increases in disease in these groups. In the United Kingdom, all patients have free access to National Health Service treatment, but new immigrants, asylum seekers, refugees, and illegal immigrants may have difficulty accessing health services because of cultural and linguistic barriers (24). Some may have difficulty in obtaining free prescriptions because of lack of necessary documents (24). In addition, immigration policies differ: in the United States, immigrants are screened and treated for active disease before entering the country, and skin-test-positive new entrants are usually treated for latent infection (25). In the United Kingdom, a few entrants are screened at airports, with the rest being screened at the local level. In some areas, only a few new entrants are screened, and chemoprophylaxis is rarely used except in children (24,26). Thus, disease prevalence among nonwhite patients may be related to inadequate screening in the United Kingdom.

Treatment completion rates in some areas of New York were low. In 1989, for example, 40% of patients with TB did not complete treatment in the city (27), and in some locales, up to 90% of patients did not complete treatment (2). Early in the epidemic, directly observed therapy was rarely used. In London, the number of patients who complete treatment is not known, and directly observed therapy is rarely used (24). An audit in a London hospital showed that 19% of patients were lost to follow-up before completing treatment. In another 15%, patient records were unavailable (A. Pearson, pers. comm.). A study of TB among the homeless in London found that 43% of suspected cases were lost to follow-up even before the diagnosis could be confirmed (23). Currently, treatment completion rates are not routinely monitored in London.

In the United Kingdom, section 37 of the Public Health (Control of Disease) Act of 1984 allows detention of patients who pose a serious risk for infection to others. In contrast to New York City, where coercion was considered by many to be an important element of TB control (28), this power is rarely used in London (24). Use of detention is difficult to justify when the infrastructure to allow less coercive adherence-support methods (such as directly observed therapy) is not in place (29). Increasing use of incentives and flexible provision of directly observed therapy in New York City has greatly decreased the use of detention (28). Directly observed therapy to improve compliance is rarely used in London, and for the least compliant patients it is almost impossible to provide (24).

Early in the epidemic in New York, there were insufficient measures to prevent spread of TB in hospitals, but by 1997 the city had 400 negative-pressure isolation facilities (30). In 1995 in London, there were 103 negative-pressure isolation facilities, only 17 of which had continuous air-pressure monitoring and 49 were considered adequate for housing patients with infectious multidrug-resistant TB (A. Hayward, unpub. obs.). Guidelines have recently been published to improve infection control in hospitals (31), but adherence will be difficult without an increase in the availability of isolation facilities.

Inappropriate treatment of isoniazid-resistant patients (e.g., failing to use an initial regimen including at least four drugs) is thought to have contributed to the development of multidrug-resistant TB in New York (12), but the explosive rise in rates of multidrug-resistant disease was mainly due to transmission of disease among hospitalized HIV-infected patients (14). It is not known what proportion of patients in London are initially treated with a four-drug regimen. The high rates of isoniazid resistance in London indicate the potential for rates of multidrug-resistant TB to increase if such regimens are not used. Hospital transmission could then lead to further rapid increases.

At the beginning of the epidemic in New York, surveillance did not provide routine data on drug resistance, ethnicity, country of origin, HIV-related TB, or treatment completion rates. The lack of information delayed recognition of the problem, and assumptions that TB was an immigrant problem proved not to be accurate (32). Similarly, surveillance in London has failed to collect routine data. Drug resistance levels have been monitored routinely only since 1994, and information (such as knowledge of previous treatment) that would be of value in interpreting routine data is often not available. An enhanced surveillance system has been launched in the United Kingdom, but many London districts are having difficulties in obtaining the additional information required (24). The new system does not currently collect data on treatment completion rates, but collecting such data is proposed for the next stage of development (J. Watson, pers. comm.).

During the early part of the TB epidemic in New York City, TB control was underfunded and highly fragmented (3). Underfunding of TB services is also a major barrier to improving TB control in London (24), and the system is also highly fragmented (24). For example, in London in 1993, TB was treated by 250 doctors working in 45 different hospitals (5). In the United Kingdom, district-based "Consultants in Communicable Disease Control" are responsible for control of TB and other infectious diseases in the community. Unlike TB coordinators in the United States, they have no direct authority to design treatment programs or institute directly observed therapy.

### Conclusions

The epidemiology and control of TB in London now differ in several ways from those in New York City early in the epidemic: London has a lower proportion of cases in HIV-infected patients, a higher proportion of cases among immigrants, and lower levels of multidrug resistance. However, similarities in epidemiology and control include similar numbers of cases; a similar increase in disease prevalence; very high prevalence in areas of lower socioeconomic conditions and large ethnic populations; concentrations of disease in the homeless, the HIV infected, and immigrants; transmission of multidrug-resistant strains in hospitals; inadequate isolation facilities; unknown treatment completion rates; high levels of loss to follow-up; and no overall coordination among providers of TB services. All these factors suggest that if TB control in London is not improved, the city could experience an epidemic of similar proportions to that in New York.

Although need for action was recognized in New York City in 1987, a clear plan for action was not implemented until 1992. By 1995, the epidemic had cost more than $1 billion (3). London needs to learn from the New York epidemic and take prompt action to improve control by developing solutions based on the local epidemiology of the disease.

### Acknowledgment

This study was conducted at the Public Health Laboratory Service, Communicable Disease Surveillance Centre, London Region, United Kingdom.

Dr. Hayward is a Lecturer in Public Health and Epidemiology at the University of Nottingham. His research interests are the epidemiology and control of infectious diseases, particularly tuberculosis.

Dr. Coker is affiliated with the Imperial College School of Science, Technology and Medicine in London as a Senior Lecturer. His book, "From Chaos to Coercion: Detention and the Control of Tuberculosis," is to be published in January 2000, by St. Martin's Press of New York.

### References

1. World Health Organization. Tuberculosis—a global emergency. WHO report on the TB epidemic. Geneva: The Organization; 1994.
2. Brudney K, Dobkin J. Resurgent tuberculosis in New York City: human immunodeficiency virus, homelessness and the decline of tuberculosis control programs. American Review of Respiratory Disease 1991;144:745-9.
3. Frieden TR, Fujiwara PI, Washko RM, Hamburg MA. Tuberculosis in New York City—turning the tide. N Engl J Med 1995;333:229-33.
4. Bureau of Tuberculosis Control Information Summary 1998. New York: New York City Department of Health; 1999.
5. Hayward AC, Watson JM. Tuberculosis in England and Wales 1982-1993: notifications exceeded predictions. Commun Dis Rep Wkly 1995;5:RR29-33.
6. Hayward A. Tuberculosis control in London: the need for change. London: NHS Executive; 1998.
7. Tuberculosis in New York City, 1988. New York: New York City Department of Health; 1990.
8. Information summary. New York City: Bureau of Tuberculosis Control, New York City Department of Health, 1995.
9. Ormerod LP, Charlett A, Gilham C, Darbyshire JH, Watson JM. Geographical distribution of tuberculosis in national surveys of England and Wales in 1998 and 1993: report of the Public Health Laboratory Service, British Thoracic Society, Department of Health Collaborative Group. Thorax 1998;53:176-81.
10. Hayward AC, Goss S, Drobniewski F, Saunders N, Goyal M, Shaw R, et al. Molecular epidemiology of tuberculosis in London. British Thoracic Society Winter Conference, December 1997.
11. Marshall BG, Mitchell DM, Shaw RJ, Marais F, Watkins RM, Coker RJ. HIV and tuberculosis coinfection in an inner London Hospital—a prospective anonymized seroprevalence study. Journal of Infection 1999:38:162-6.
12. Frieden TR, Sterling T, Pablos-Mendez A, Kilburn JO, Cauthen GM, Dooley SW. The emergence of drug-resistant tuberculosis in New York City. N Engl J Med 1993;328:521-6.
13. Irish C, Herbert J, Bennett D, Gilham C, Drobniewski F, Williams R, et al. Database study of antibiotic resistant tuberculosis in the United Kingdom, 1994-6. BMJ 1999;318:497-8.
14. Frieden TR, Woodley CL, Crawford JT, Lew D, Dooley SM. The molecular epidemiology of tuberculosis in New York City: the importance of nosocomial transmission and laboratory error. Tuber Lung Dis 1996;77:407-13.

15. Outbreak of hospital acquired multidrug-resistant tuberculosis. Commun Dis Rep CDR Wkly 1994;4:1.
16. Breathnach AS, de Ruiter A, Holdsworth GM, Bateman NT, O'Sullivan DG, Rees PJ, et al. An outbreak of multidrug resistant tuberculosis in a London teaching hospital. J Hosp Infect 1998;39:111-7.
17. Kent PJ, Uttley AHC, Stoker NG, Miller R, Pozniak AL. Transmission of tuberculosis in British centres for patients infected with HIV. BMJ 1994;309:639-40.
18. Citron KM, Southern A, Dixon M. Out of the shadow. London: CRISIS; 1995.
19. Dugger CW. Study says shelter turnover hides scope of homelessness. The New York Times; 1993 November 16:A1.
20. Sherman MN, Brickner MS, Schwartz MS. Tuberculosis in single-room occupancy hotel residents: a persisting focus of disease. New York Medical Journal 1980;2:39-41.
21. McAdam JM, Brickner PW, Scharer LL, Crocco JA, Duff AE. The spectrum of tuberculosis in a New York City men's shelter clinic (1982-1988). Chest 1990;97:798-805.
22. Tuberculosis in New York City, 1992. New York City: New York City Department of Health; 1992.
23. Kumar D, Citron KM, Leese J, Watson JM. Tuberculosis among the homeless at a temporary shelter in London: report of a chest X-ray screening programme. J Epidemiol Community Health 1995;49:629-33.
24. Results of pan London multidisciplinary workshops on tuberculosis control. Report to the Director of Public Health for London; 1999.
25. American Thoracic Society. Control of tuberculosis in the United States. Am Rev Respir Dis 1992;146:1623-33.
26. Hardie RM, Watson JM. Screening immigrants at risk of tuberculosis. BMJ 1993;307:1539-40.
27. Tuberculosis. City Health Information (CHI) 1996;15:12-3.
28. Gasner MR, Maw KL, Feldman GE, Fujiwara PI, Frieden TR. The use of legal action in New York City to ensure treatment of tuberculosis. N Engl J Med 1999;340:359-66.
29. Coker RJ. Public health, civil liberties, and tuberculosis. BMJ 1999;318:1434-5.
30. Perales, CA. Memorandum to Mayor David N. Dinkins. Update on tuberculosis control activities. New York: Office of the Mayor; 1998.
31. The Interdepartmental Working Group on Tuberculosis. The prevention and control of tuberculosis in the United Kingdom. Prevention of transmission of drug resistant tuberculosis and tuberculosis in HIV infected patients. London: Her Majesty's Stationery Office; 1999.
32. Bellin E. Failure of tuberculosis control: a prescription for change. JAMA 1994;271:708-9.
